# TRBC1/TRBC2 RNA In Situ Hybridization as a Diagnostic Approach for Canine and Feline T-Cell Lymphoma: A Proof-of-Concept Study

**DOI:** 10.3390/vetsci13040330

**Published:** 2026-03-28

**Authors:** Honoria M. E. Brown, Jonathan J. Wilson, Daniel Rodgers, Shelley C. Evans, Julia Jones, Jianxiong Pang, Joy Archer, Fernando Constantino-Casas, Sam Parsons, Adam G. Scott, Anuradha Kaistha, Elizabeth J. Soilleux

**Affiliations:** 1 Department of Pathology, University of Cambridge, Tennis Court Road, Cambridge CB2 1QP, UK; drhonoriabrown@gmail.com (H.M.E.B.); jw35778@gmail.com (J.J.W.); sce30@cam.ac.uk (S.C.E.); jianxiong.pang@crick.ac.uk (J.P.); sam.parsons@nhs.net (S.P.); as3547@cam.ac.uk (A.G.S.); ak2140@cam.ac.uk (A.K.); 2 Department of Veterinary Medicine, University of Cambridge, Madingley Road, Cambridge CB3 0ES, UK; jra11@cam.ac.uk (J.A.); fc307@cam.ac.uk (F.C.-C.); 3 Human Research Tissue Bank, Cambridge University Hospitals NHS Foundation Trust, Hills Road, Cambridge CB2 0QQ, UK; daniel.rodgers@nhs.net; 4 Cancer Research UK Cambridge Institute, Li Ka Shing Centre, University of Cambridge, Cambridge CB2 0RE, UK; julia.jones@cruk.cam.ac.uk

**Keywords:** clonal, T-cell receptor (TCR), transcript, monotypia, hematopathology, veterinary, simple, rapid, definitive, cost-saving

## Abstract

Background/Objectives: T-cell lymphomas are relatively common in veterinary species, yet current diagnostic tools such as PCR-based clonality assays often lack sensitivity and specificity. In humans, we recently developed two related tissue-based diagnostic approaches based on the differential detection of the mutually exclusively expressed TCRbeta1 and 2 (TCRβ1 and 2) constant region proteins, or the corresponding *TRBC1* and *TRBC2* transcripts. Analogous to the detection of kappa/lambda light chains for the diagnosis of B-cell/plasma cell neoplasms in human clinical practice, our TCRβ1/2 diagnostic assay has the potential to transform veterinary diagnostic workflows. Methods: We identified and confirmed the sequences of the relevant *TRBC1* and *TRBC2* sequences in both cats and dogs, focusing on the 3′ untranslated region (UTR), where there is the least sequence homology between *TRBC1* and *TRBC2*. To allow us to design appropriate probe sequences, we confirmed a lack of 3′UTR in either species, and we observed limited 3′ untranslated region UTR sequence polymorphism in the cat but not in the dog 3′UTR. We designed BaseScope™ RNA in situ hybridization probes targeting the 3′ UTR to distinguish between *TRBC1* and *TRBC2* transcripts in formalin-fixed paraffin-embedded tissues. Results: In normal tissues, we found the *TRBC2*:*TRBC1* expression ratio to be similar to the 1.2:1 ratio in humans, between 1:1 and 3:1, skewing towards *TRBC2*, in both dogs and cats. These findings were corroborated using quantitative reverse transcription PCR. Applying our in situ hybridization probes to cases of T-cell lymphoma in dogs and cats, we demonstrated that an assay for differential expression of *TRBC1* and *TRBC2* in T-cell populations could identify clonal T-cell populations, as in human diagnostics. If further studies corroborate this proof-of-concept study, TRBC1/2 detection could obviate the need for slow, complex and expensive multiplexed PCR-based (PCR for antigen receptor rearrangements (PARR)) clonality assays. Conclusions: This study provides proof-of-concept data for a novel diagnostic approach that could simplify and substantially improve the accuracy of lymphoma diagnostics in veterinary medicine, by detecting *TRBC1*/*2* transcripts.

## Simple Summary

T-cell lymphomas are a relatively common tumor of white blood cells in cats and dogs, yet current diagnostic tools are slow, expensive and not entirely accurate. Presently, DNA must be extracted from the suspected lymphoma and subjected to molecular testing, which can give inconclusive or incorrect results under certain circumstances. The situation is similar in human T-cell lymphoma diagnosis, but we have recently developed an assay that can give more rapid, cheap and accurate diagnosis. Our aim was to develop an analogous assay for cats and dogs and to test it in clinical veterinary specimens. The assay depends on detecting two closely related but distinct targets in T-cells. Each T-cell will have only one of these targets. If a population of T-cells is a roughly equal mixture of cells with the two target types, they are benign. If most or all of the T-cells have the same target type, they are likely to be lymphoma. To achieve this, we had to first check the sequences of the targets and then develop probes to detect RNA from the two targets. We applied this assay to clinical specimens and showed, in a very small number of cases, that this assay has the potential to be used in clinical practice to distinguish T-cell lymphomas from benign T-cell populations. This work has the potential to make the diagnosis of T-cell lymphoma in cats and dogs simpler, faster and more accurate, as well as decreasing cost, which may make accurate diagnosis of T-cell lymphoma a reality for a wider range of pet owners.

## 1. Introduction

T-cell lymphoma represents a clinically significant malignancy in small animal practice, presenting most frequently as enlarged lymph nodes or organomegaly. Lymphoid and myeloid tumors together represent around one third of all feline cancers, and an estimated 200 cases of lymphoid neoplasia are seen per 100,000 domestic cats (*Felis catus*) [1]. A feline lymphoma immunophenotyping study reported that 49% of lymphomas were T-cell, 25% B-cell and 26% of undetermined type [2]. In domestic dogs (*Canis lupus familiaris*), lymphoma accounts for 7% to 24% of all malignancies, with T-cell lymphoma comprising approximately 30–40% of these [1,3]. There is significant variation between breeds, with some, such as Shih Tzu and Siberian Husky, displaying significant (>80%) preponderance toward the T-cell lymphoma subtype [4].

Despite the prevalence of this disease in small animals, diagnostic pathways in T-cell lymphoma remain suboptimal. Cytological examination of fine-needle aspirates from lymph nodes is rapid, minimally invasive and reasonably specific for the diagnosis of high-grade lymphoma [5]. However, it lacks sensitivity for other lymphoma types, such as mesenteric T-cell lymphoma [6]. Flow cytometry remains a valuable diagnostic tool in practice for determining lymphoid lineage, but it cannot reliably distinguish reactive from neoplastic lymphoid populations [3]. Surgical biopsy for histomorphological examination combined with immunohistochemistry is the gold standard, but it can also be inconclusive, particularly when samples lack overt morphological evidence of neoplasia [7]—for example, in differentiating feline type II enteropathy-associated T-cell lymphoma (EATL) from inflammatory bowel disease (IBD) [8,9].

In cases in which histomorphological and immunohistochemical examination cannot definitely confirm or exclude T-cell lymphoma, polymerase chain reaction (PCR) for antigen receptor rearrangements (PARR) may be performed [10]. This technique uses multiplexed PCR reactions to amplify the variable regions of the T-cell receptor (TCR) genes. The presence of one or more exaggerated peak(s) on an electropherogram indicates a single over-represented amplicon size, suggesting a clonal (also known as monoclonal) T-cell population and likely T-cell lymphoma [10]. Similar challenges are seen in human clinical practice and such cases are sent for analogous multiplexed PCR-based clonality studies [11]. In both clinical and veterinary practice, these PCR assays lack morphological context, are expensive, are limited to specialist centers and can delay diagnosis due to extended turnaround times. They can also be difficult to interpret or give false positives [11,12,13,14].

We recently described a novel approach for human clinical samples to distinguish monoclonal from polyclonal populations of T-cells in tissue sections without DNA extraction [11,14]. Our novel approach has the potential to revolutionize diagnostic workflows in tissue sections. To do this, we detect the two T-cell receptor beta constant regions, TCRbeta1 and TCRbeta2, which show mutually exclusive expression at the individual cellular level. All T-cells in a clonal population express the same TCRbeta type, while polyclonal T-cell populations show a roughly equal mixture of the two types [11,14]. This approach, analogous to the diagnostic use of kappa/lambda (κ/λ) light chain restriction in human B-cell and plasma cell neoplasia, offers substantial advantages for the diagnosis of T-cell malignancies, including the ability to simultaneously assess the architecture, cytomorphology and (on serial sections) the immunophenotype of a suspected clonal T-cell population [11,14]. To date, no such reagents exist for veterinary species. This is a proof-of-concept study for a parallel diagnostic tool for use in canine and feline pathology.

As in humans, in adult dogs, alpha-beta T-cells predominate (~97.5%, across breeds) over gamma-delta T-cells, with comparable percentages in cats [15,16]. Nearly all T-cell malignancies in these species arise from alpha-beta T-cells, with gamma-delta-derived tumors being exceptionally rare [17,18], analogous to the situation in humans [19].

**Figure 1:**
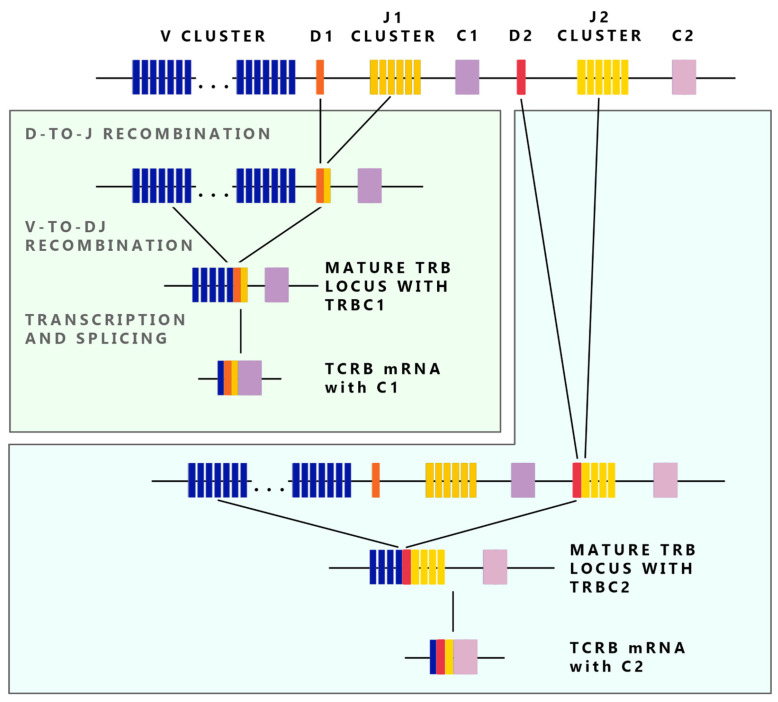
During T-cell development, antigen receptor diversity arises through somatic V(D)J recombination, a process mediated by the recombinase enzymes RAG-1 and RAG-2. These endonucleases initiate cleavage at recombination signal sequences, enabling the assembly of variable (V), diversity (D) and joining (J) gene segments. There are 33 known TRBV genes in the cat and 36 in the dog, with both species sharing the same number of J (12), D (2) and C (2) genes in the *TRB* locus [21,22,23]. Junctional diversity is further amplified by exonucleolytic trimming, palindromic nucleotide additions and random nucleotide insertions (via terminal deoxynucleotidyltransferase, TdT). It is this process that produces a vast repertoire of unique T-cell receptors (greater than 25 × 106) capable of recognizing an enormous number of antigens [24].

During thymic development, TCR genes undergo somatic recombination through a mostly stochastic process involving the selection of variable (V), diversity (D) (for the beta and delta chains only) and joining (J) gene segments, along with random insertions or deletions at the junctions (Figure 1). A previously underappreciated feature of the TCR beta (*TRB*) locus is the presence of two distinct constant (C) regions that are the target of our approach. The germline *TRB* locus contains a cluster of V genes (*TRBV*) upstream of two D-J-C clusters, *TRBD1-TRBJ1-TRBC1* and *TRBD2-TRBJ2-TRBC2* (Figure 1). Recombination proceeds sequentially, with D-to-J joining followed by V-to-DJ rearrangement. The key consequence for this study is that the rearranged TCR sequence will contain either *TRBC1* or *TRBC2*, but not both. Although rearrangement can occur on both chromosomes, leading to a fully rearranged *TRBC1* on one chromosome and a fully rearranged *TRBC2* on the other, one of the two rearrangements is usually non-functional (i.e., it contains a stop codon) [20], and thus TCRbeta (1 or 2) protein isotype expression is mutually exclusive in individual T-cells.

To provide proof-of-concept for a veterinary T-cell lymphoma diagnostic based on T-cell monotypia, we identified and characterized the *TRBC1* and *TRBC2* sequences in both cats and dogs, focusing on the 3′ untranslated regions (3′ UTRs). We found these transcripts to be intron-free, with limited polymorphism present in the feline but not the canine sequence. We designed BaseScope^TM^ probes targeting these sequences and applied them to normal lymphoid tissues, establishing baseline, quantitative PCR-corroborated *TRBC2*:*TRBC1* expression ratios of between 1:1 and 3:1 in both dogs and cats, not dissimilar to the 1.2:1 ratio in humans [11,14]. Finally, we applied these probes to a very small number of samples of feline and canine T-cell lymphoma and demonstrated that T-cell monotypia (*TRBC*1/2 restriction) might serve as a surrogate for clonality in cats and dogs if further larger studies corroborate our findings.

## 2. Materials and Methods

### 2.1. In Silico Prediction of Cat and Dog TRBC1 and TRBC2 Sequences

**Figure A1:**
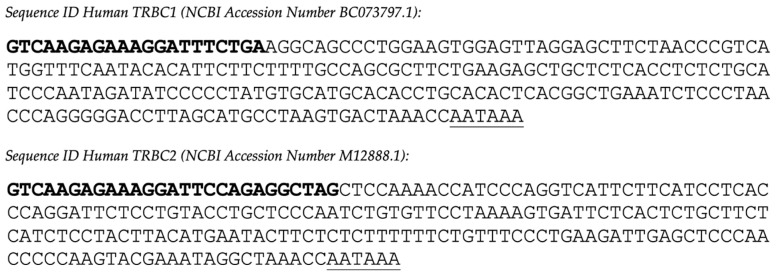
Human sequences used to search databases for Cat and Dog TRBC1/2 sequences. Bold font represents the end of the coding sequence (CDS), and underlined sequence represents the polyadenylation site.

The last 21 bases of the coding region for each human *TRBC* segment (*TRBC1* (Accession Code BC073797.1) and *TRBC2* (Accession Code M12888.1; Appendix A: Figure A1) were used as search queries in BLAST+ version 2.7.1 (https://blast.ncbi.nlm.nih.gov) to search the NCBI non-redundant/nucleotide (nr/nt) collection and databases containing sequence read archives (SRA) (http://www.ncbi.nlm.nih.gov/Traces/sra (accessed on 15 October 2018)), whole-genome shotgun contigs (wgs) (https://www.ncbi.nlm.nih.gov/genbank/wgs/ (accessed on 16 October 2018)) and expressed sequence tags (est) (https://www.ncbi.nlm.nih.gov/genbank/dbest/ (accessed on 17 October 2018)). Each consensus was constructed starting from a point at least 300 base pairs (bp) 5′ of the end of the coding sequence (CDS) and ending at the polyadenylation site. Predicted *TRBC1* and *TRBC2* sequences for the cat and dog genera were assembled in CodonCode Aligner version 7.1.2 (CodonCode Corporation, Centerville, MA, USA). Sequence data was uploaded into CodonCode Aligner via GenBank accession codes and compiled using end-to-end alignment.

### 2.2. PCR and Sequencing-Based Confirmation of Cat and Dog TRBC1 and TRBC2 Sequences

To confirm the predicted sequences, samples of cat, dog and human (used as control) cDNA produced with a poly (dT) primer were obtained from Amsbio (Dog DD-307, Cat FD-307 and normal Adult Human C1234226). *TRBC1* and *TRBC2* sequences were amplified by PCR using the AmpliTaq Gold 360 Master Mix (Life Technologies, Paisley, UK, 4398876) on a Tetrad 2 cycler (Bio-Rad, Watford, UK). Samples were separated on a 2.5% agarose gel (Invitrogen Life Technologies, 3105678) using GelPilot loading dye (Qiagen, Manchester, UK, 127132603) and were visualized with 2.4 μL of 10 mg/mL ethidium bromide solution (Sigma-Aldrich, Haverhill, UK, 075K8917). Hyperladder IV (Bioline, London, UK, H4-106F) was loaded as a size standard. Primers (Sigma Aldrich) were as follows: Dog *TRBC1* Forward: CTACTGTCTGAGCAGCCGGC, Reverse: GTCACACTAGGGACCCCC; Dog *TRBC2* Forward: CTACTGTCTGAGCAGCCGGC, Reverse: TCAGGCTTGAGGAGCTCAGTC; Cat *TRBC1* Forward: CTACTGTCTGAGCAGCCGGC, Reverse: AGTCAATTTCGTCACGCGAGG; Cat *TRBC2* Forward: CTACTGTCTGAGCAGCCGGC, Reverse: AGCTCAATCCACAGGGAAGTG; Human *TRBC1* Forward: ATACTGCCTGAGCAGCCGC, Reverse: GCTAAGGTCCCCCTGGGTTAG; Human *TRBC2* Forward: ATACTGCCTGAGCAGCCGC, Reverse: GCCTATTTCGTACTTGGGGG. All PCR products were confirmed by Sanger sequencing (Department of Biochemistry, The University of Cambridge) and SnapGene version 4.1.3 (www.snapgene.com) was used to compare the sequencing data.

### 2.3. Investigation of Level of Polymorphism in Cat and Dog TRBC1 and TRBC2 Sequences

To investigate the level of 3′ UTR polymorphism, cat and dog EDTA blood samples were randomly selected from the sample archive at the Queens’ Veterinary School Hospital (University of Cambridge, Cambridge, UK). Genomic DNA was extracted using the DNeasy Blood and Tissue kit (Qiagen, 69504) according to the manufacturer’s instructions and the concentration was determined using NanoDrop (Bioline ND-1000 spectrophotometer). PCR was performed using the primers described above. PCR products were subjected to Sanger sequencing (Department of Biochemistry, University of Cambridge, Cambridge, UK). SnapGene 4.1.3 was used to analyze the sequencing data by means of alignment with the consensus sequences, allowing potential read errors to be identified, and supplemented by manual interpretation as required. A high-stringency filter was initially used to filter low-quality peaks from the data, and the forward and reverse Sanger sequencing reads were constructed into a single contig for each sample. These were then aligned with the reference sequence using CodonCode Aligner to identify the location of polymorphisms.

### 2.4. Investigation of Level of Polymorphism in Cat and Dog TRBC1 and TRBC2 Coding Regions

A similar approach to sequencing the coding regions was taken for cats (common *TRBC1*/*2* forward primer: ACAGGCTTCTACCCCGACCA; common *TRBC1*/*2* reverse primer: CCCACTGGTCATCCTTCCCG) and dogs (common *TRBC1/2* forward primer: GGATCTGCAGAAGGTCACCCC; common *TRBC1/2* reverse primer: GACTTGGCAGCGGAAGTGGT). Because the *TRBC1*/*2* coding region sequence identity was so high within each species, it was difficult to make isotype-specific primers. Thus, sequences corresponding to both isotypes were amplified in 10 canine and feline samples, each using AmpliTaq Gold 360 Master Mix and 360 GC enhancer (Part Number 4398876; Life Technologies) and subjected to Illumina next-generation sequencing. DNA libraries were prepared using the NEBNext^®^ Ultra™ II DNA Library Prep Kit for Illumina^®^ (NEB #E7645S/L; New England Biolabs, Hitchin, UK) in combination with NEBNext^®^ Multiplex Oligos for Illumina^®^ (Unique Dual Index UMI Adaptors DNA) (NEB #E7103S/L), following the manufacturer’s instructions. AMPure beads (Product No: A63880; Beckman Coulter) and D1000 ScreenTape (Cat. No. 5067-5582; Agilent Technologies, Cheadle, UK) were used for cleanup and quality control for NGS sequencing.

### 2.5. Comparison of Cat and Dog TRBC1 and TRBC2 Sequence Data with Reference Database Sequences

Raw sequences were aligned using minimap2 short-read preset [25] to canine and feline *TRBC1*/*2* germline reference sequences obtained from the International ImMunoGeneTics information system Database (IMGT; www.imgt.org) (Dog Accession Numbers BK065025 and HE653929; Cat, Accession Number IMGT000037). The resulting SAM files were converted into BAM files, indexed and subject to quality-control measures using SAMtools [26]. The processed BAM files were filtered by Argparse (a component of Python version 3.10, Python Software Foundation) [27] and read by Pysam version 0.23.3 [28] to determine coverage of reads at the base distinguishing TCRB1 from TCRB2 and compared to the reference sequences loaded by Biopython version 1.86 [29]. Base mismatches were recorded and written to .xlsx files using Pandas version 2.3.1 [30] (Pandas Development Team).

### 2.6. Preparation of Formalin-Fixed Paraffin-Embedded (FFPE) Cat and Dog Tissue Samples

**Figure 2:**
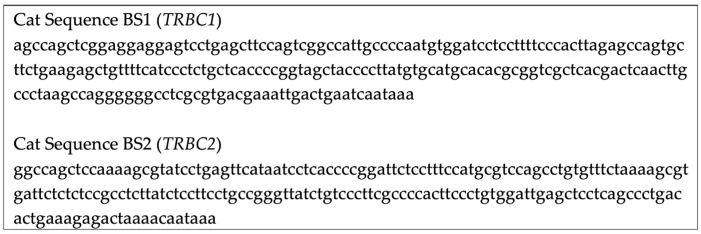
3′ UTR sequences provided to Bio-Techne for cat *TRBC1*/*TRBC2* probe design. Exact probe sites are proprietary to Bio-Techne.

**Figure 3:**
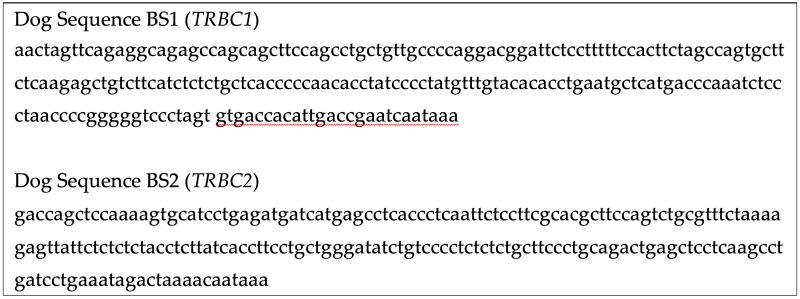
3′ UTR sequences provided to Bio-Techne for dog *TRBC1*/*TRBC2* probe design. Exact probe sites are proprietary to Bio-Techne.

T-cell lymphoma and benign lymphoid formalin-fixed paraffin-embedded (FFPE) cat and dog samples were obtained from the Cambridge University Veterinary School with institutional ethical approval. These included 7 cat samples (2 T-cell lymphomas, 5 benign) and 9 dog samples (2 T-cell lymphomas, 5 benign). All malignant cases were deemed suspicious for T-cell lymphoma on the basis of morphological and immunohistochemical examination, with a subsequent T-cell clonal result on PARR. Sections were cut at 3.5 µm and multiplexed onto positively charged slides for hematoxylin and eosin (H&E) staining. BaseScope^TM^ in situ hybridization was performed for canine/feline *TRBC1*, *TRBC2* (Figure 2 and Figure 3), positive control (*PPIB*) and negative control (*dapB*), using BaseScope^TM^ LS probes (Bio-Techne, Abingdon, UK) and the BaseScope™ LS Reagent Kit (Bio-Techne, Abingdon, UK, 323600), as per the manufacturer’s instructions, on a Leica Bond RX automated staining machine (Leica Biosystems, Newcastle, UK).

### 2.7. BaseScope^TM^ Staining of Cat and Dog FFPE Tissue Samples

Sections were baked for 1 h at 60 °C before loading onto a Bond RX automated staining machine (Leica Biosystems, Newcastle, UK). Slides were deparaffinized and rehydrated on board before pre-treatments using Epitope Retrieval Solution 2 (Leica Biosystems, AR9640) at 95 °C for 15 min and ACD Enzyme (LS Reagent kit) at 40 °C for 15 min. Probe hybridization and signal amplification were performed according to the manufacturer’s instructions. Fast red detection of each target was performed on the Bond Rx using the Bond Polymer Refine Red chromogenic detection Kit (Leica Biosystems, DS9390) according to ACD protocol. Slides were then removed from the Bond Rx and were heated at 60 °C for 1 h, dipped in Xylene and mounted using EcoMount Mounting Medium (Biocare Medical, Pacheco, CA, USA, EM897L). The slides were imaged on the Aperio AT2 (Leica Biosystems) slide scanner to create whole slide images. Images were captured at 40× magnification, with a resolution of 0.25 microns per pixel.

### 2.8. TRBC1 and TRBC2 qPCR with Cat- and Dog-Specific Primer Sets

Five FFPE-embedded 5 µm sections from each cat and dog sample were used for *TRBC1* and *TRBC2* mRNA transcript ratio analysis using qPCR. The slides were deparaffinized and RNA was extracted using the RNeasy^®^ FFPE kit (Qiagen, 73504), following the manufacturer’s instructions. Primers used for the qPCR were as follows: Dog *TRBC1* Forward: CCACTTCTAGCCAGTGCTTC, Reverse: CCACTTCTAGCCAGTGCTTC; Dog *TRBC2* Forward: GAGACCAGCTCCAAAAGTG, Reverse: CCCAGCAGGAAGGTGATAAG; Cat *TRBC1* Forward: TCCTTTTCCCACTTAGAGCC, Reverse: GGTCGCTCACGACTCAAC; Cat *TRBC2* Forward: AGTTCATAATCCTCACCCCG, Reverse: GGGCGAAGGGACAGATAAC; Cat/dog GAPDH Forward: ATCCCGCCAACATCAAATG, Reverse: CAGAGATGATGACCCTCTT. qPCR was performed using the iTaq Universal SYBR^®^ Green Supermix (Bio Rad, 1725120) on a QuantStudio™ 6 Flex Real-Time PCR System (Catalog No. 4485697, Applied Biosystems™). The *TRBC2*:*TRBC1* expression ratio was calculated by the Delta-Delta Ct (2^#∆∆%&^) method [31]. As the comparative analysis was all performed in the same tissue at the same time, the Ct value of the internal control, glyceraldehyde-3-phosphate dehydrogenase (GAPDH), remained the same, and the calculation was simplified as follows:

Expression ratio (*TRBC2*:*TRBC1*) = 2^−ΔΔCt^

where

ΔC_t_ = C_t,target_−C_t,reference_ (Note that “target” refers to either *TRBC1* or *TRBC2*) and so

ΔΔC_t_ = ΔC_t,_*_TRBC_*_2_−ΔC_t,_*_TRBC_*_1_

## 3. Results

To develop transcript-based diagnostic tools for T-cell lymphoma in veterinary species, we needed to identify suitable sequences for probe design. We first characterized the canine and feline *TRBC1* and *TRBC2* sequences, then established baseline expression patterns in normal tissues and finally validated the diagnostic utility in confirmed lymphoma cases. Our approach focused on the 3′ untranslated regions (3′ UTRs) as probe targets, as these showed much greater sequence divergence between the *TRBC1* and *TRBC2* isotypes than the highly conserved *TRBC1*/*2* coding regions.

### 3.1. Confirmation of Sequences and Intronic Structures Shown in the International Immunogenetics Database (IMGT)

**Figure A2:**
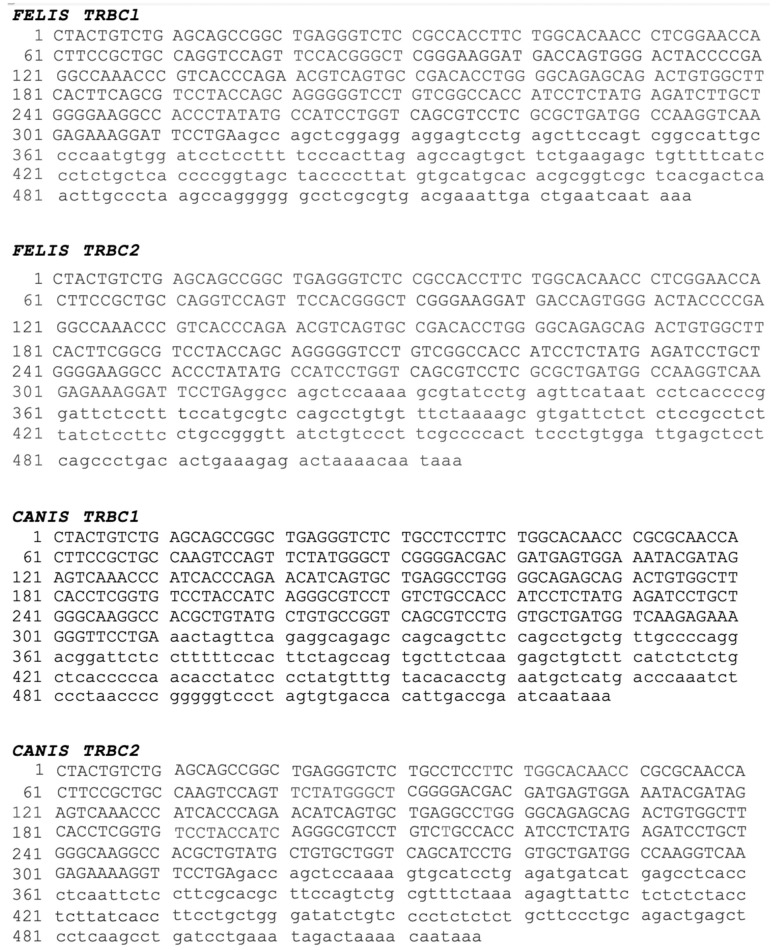
Consensus dog and cat TRBC1 and TRBC2 sequences, confirmed by PCR amplification and Sanger sequencing (CDS in capitals; 3′ UTR in lower case).

**Figure A3:**
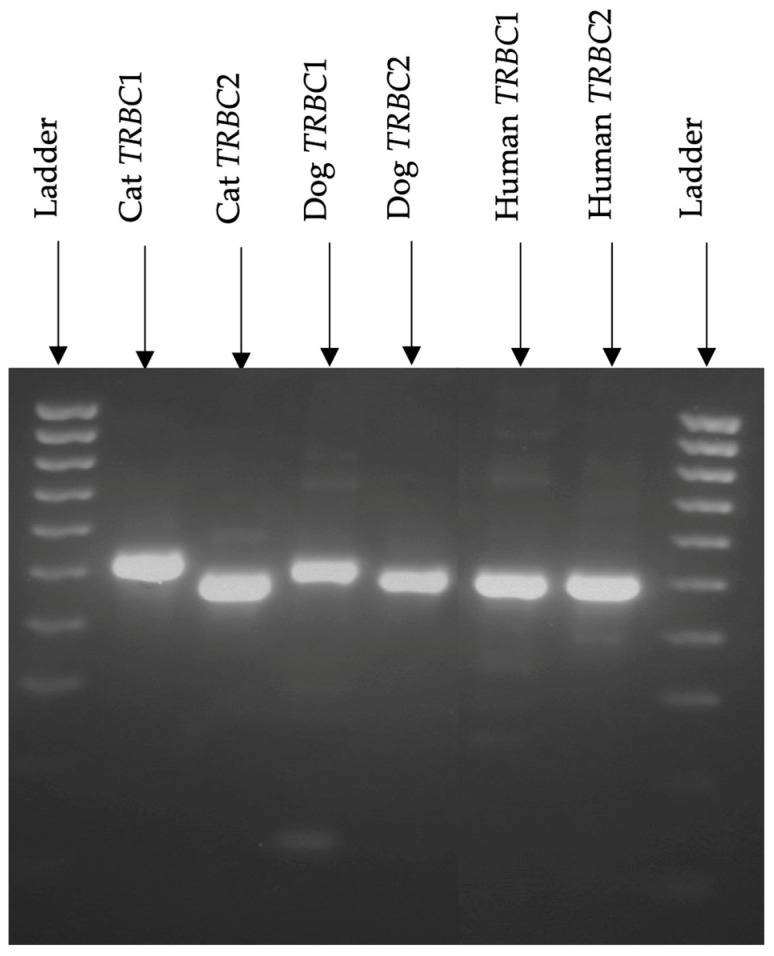
Gel showing fragments produced using common forward primers and specific reverse primers for TRBC1 and TRBC2.

To build cat and dog consensus sequences, the last 21 bases of the coding region of human *TRBC1* (*Accession Code BC073797.1*) and *TRBC2* (*Accession Code M12888.1*) (Appendix A: Figure A1) were used as the initial query sequences in BLAST searches of the NCBI non-redundant/nucleotide (nr/nt) collection, databases containing sequence read archives (SRA) (http://www.ncbi.nlm.nih.gov/Traces/sra (accessed on 15 October 2018)), whole-genome shotgun contigs (wgs) (https://www.ncbi.nlm.nih.gov/genbank/wgs/ (accessed on 16 October 2018)) and expressed sequence tags (est) (https://www.ncbi.nlm.nih.gov/genbank/dbest/ (accessed on 17 October 2018)), identifying sequences shown in Appendix A: Table A1. Each consensus sequence (cat *TRBC1*, cat *TRBC2*, dog *TRBC1*, dog *TRBC2*) was constructed starting from a point at least 300 base pairs (bp) 5′ of the end of the coding sequence (CDS) and ending at the polyadenylation site (Appendix A: Figure A2). The consensus sequences were used to design primers for *TRBC1* and *TRBC2* amplification from cat and dog cDNA. Amplification of *TRBC1* and *TRBC2* produced fragments of lengths corresponding to those expected from the in silico sequences (Appendix A: Figure A3). Sanger sequencing of these fragments confirmed the identity of the predicted 3′ UTR sequences with those in the International Immunogenetics Database (IMGT; www.imgt.org) (IMGT000037 (*Felis catus*), IMGT000005 (*Canis lupus familiaris*)) and comparison with the IMGT genomic sequence also confirmed a lack of introns within the 3′ UTR.

The *TRBC1* and *TRBC2* coding regions are highly conserved (99% nucleic acid identity in the cat; 97% in the dog). This makes them a poor target for probe design. However, the 3′ UTRs were found to be highly divergent (58% *TRBC1*/*TRBC2* identity in the cat; 55% *TRBC1*/*TRBC2* in the dog). Probe design against the 3′ UTR therefore permitted differential *TRBC1*/*2* transcript detection in both species, with chromogenic in situ hybridization (CISH)-based detection, using BaseScope^TM^ (Bio-Techne, Abingdon, UK).

### 3.2. TRBC1 and TRBC2 3′ UTR Polymorphisms Are Seen in Cats but Not Dogs

**Figure 4:**
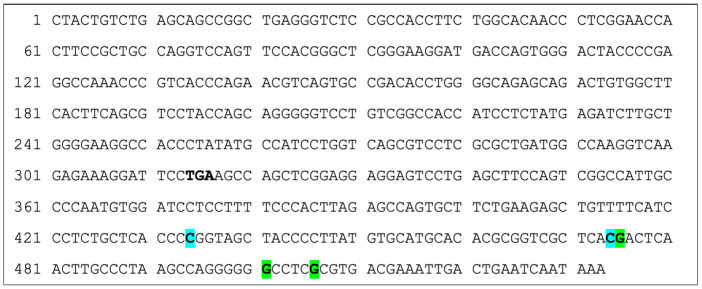
Polymorphisms in the Cat *TRBC1* including 3′ UTR (533 bp ds-DNA; CDS: 1-316; 3′ UTR: 317-533): TGA (bold) signifies end of coding region. **G** (highlighted green) signifies G > A, and **C** (highlighted turquoise) signifies C > T. Out of 20 cats, polymorphisms were found at the following positions in *TRBC1*: 434, 474, 475, 500, 505. At position 434, 16 (80%) cats were homozygotic for cytosine (C), 3 (15%) cats were heterozygotic for cytosine (C) and thymine (T), and 1 (5%) cat was homozygotic for thymine (T). At position 474, 19 (95%) cats were homozygotic for cytosine (C), and 1 (5%) cat was heterozygotic for cytosine (C) and thymine (T). At position 475, 16 (80%) cats were homozygotic for guanine (G), and 4 (20%) cats were heterozygotic for guanine (G) and adenine (A). At position 500, 11 (55%) cats were homozygotic for guanine (G), 7 (35%) cats were heterozygotic for guanine (G) and adenine (A) and 2 (10%) cats were homozygotic for adenine (A). At position 505, 18 (90%) cats were homozygotic for guanine (G), and 2 (10%) cats were heterozygotic for guanine (G) and adenine (A). Details of the breeds of cat are included in Table 2.

**Figure 5:**
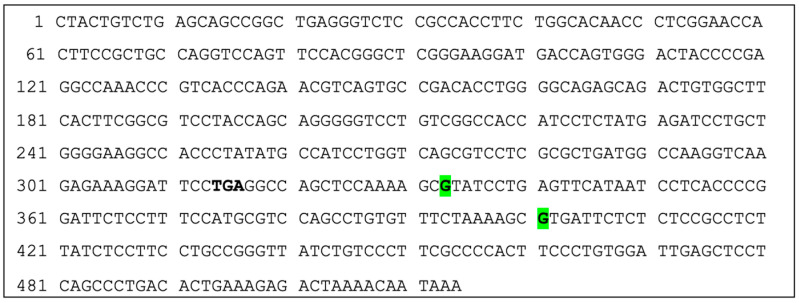
Polymorphisms in cat *TRBC2* including 3′ UTR (514 bp ds-DNA; CDS: 1-316; 3′ UTR: 317-514): TGA (bold) signifies end of coding region. **G** (highlighted green) signifies G > A. Out of 20 cats, polymorphisms were found at the following positions in *TRBC2*: 333 and 400. At position 333, 18 (90%) cats were homozygotic for guanine (G), and 2 (10%) cats were heterozygotic for guanine (G) and adenine (A). At position 400, 14 (70%) cats were homozygotic for guanine (G), 4 (20%) cats were heterozygotic for guanine (G) and adenine (A) and 2 (10%) cats were homozygotic for adenine (A).

In addition to requiring sufficient sequence divergence for differential *TRBC1*/*2* detection, an in situ hybridization assay will only be successful if there is minimal or no polymorphism at the probe binding sites, with any single-nucleotide polymorphisms (SNPs) decreasing probe sensitivity due to less efficient binding. We amplified and sequenced *TRBC1* and *TRBC2* 3′ UTRs from multiple individual animals to inform probe design. No *TRBC1* or *TRBC2* 3′ UTR sequence polymorphisms were detected in samples from 10 dogs (breeds detailed in Table 1). However, in the 20 cat samples (breeds detailed in Table 2), single-nucleotide polymorphisms (SNPs) were identified in both *TRBC1* (at 5 separate locations) and *TRBC2* (at separate 2 locations) 3′ UTRs (Figure 4 and Figure 5, respectively). The various SNPs were present in between 1 (5%) and 9 (45%) cats. Additionally, some samples contained multiple independent SNPs.

**Table 1:** BaseScope and qPCR results for dog T-cell lymphoma and benign lymphoid FFPE samples.

Case Number	Breed	Sex (M = Male; F = Female)	Age (Years and Months)	Sample	Diagnosis	qPCR mRNA Level *TRBC1*: *TRBC2*	BaseScope^TM^ Estimated *TRBC1*: *TRBC2* Ratio (and *TRBC2*: *TRBC1* Ratio)	BaseScope^TM^ Comments
Dog 1	Springer spaniel	M	7Y 10M	Colonic lymph node	Benign	3.65:1	0.67:1 (1.5:1)	Cell numbers relatively similar, but some *TRBC1*+ cells more intensely stained.
Dog 2	Springer spaniel	F	12Y	Pharyngeal lymph node	Benign	2.95:1	1:1 (1:1)	
Dog 3	Labrador	M	7Y 5M	Gastric lymph node	Benign	4.49:1	0.5:1 (2:1)	
Dog 4	West highland white terrier	M	11Y 1M	Colonic lymph node	Benign	1.55:1	0.33:1 (3:1)	
Dog 5	Golden retriever	M	5Y 3M	Prescapular lymph node	Benign	2.72:1	0.67:1 (1.5:1)	
Dog 6	Golden retriever	F	8Y 6M	Skin	T-cell lymphoma	11.8:1	5:1 (0.2:1)	*TRBC1*-restricted.
Dog 7	Beagle	M	10Y	Mandibular lymph node	T-cell lymphoma	1.12:1	0.125:1 (8:1)	*TRBC2*-restricted. Widespread weakly *TRBC2*+ cells with occasional strongly stained *TRBC1*+ and *TRBC2*+ tumor-infiltrating benign T-cells, but the *TRBC1*+ T-cells are markedly more strongly stained than the *TRBC2*+ cells.

**Table 2:** BaseScope and qPCR for cat T-cell lymphoma and benign lymphoid FFPE samples.

Case Number	Breed	Sex (M = Male; F = Female)	Age (Years and Months)	Sample	Diagnosis	qPCR mRNA Level *TRBC2*: *TRBC1*	BaseScope^TM^ Estimated *TRBC2*: *TRBC1* Ratio	BaseScope^TM^ Comments
Cat 1	Main coon	M	1Y 5M	Mesenteric lymph node	Benign	1.81:1	2:1	
Cat 2	Domestic shorthair	M	6Y 2M	Sternal lymph node	Benign	6:1	2:1	
Cat 3	Domestic shorthair	M	12Y	Cervical lymph node	Benign	3.99:1	3:1	
Cat 4	Domestic shorthair	F	8M	Mesenteric lymph node	Benign	3.16:1	3:1	
Cat 5	Domestic shorthair	M	14Y 5M	Scapular lymph node	Benign	4.38:1	1:1	
Cat 6	Russian blue	F	12Y 6M	Mesenteric lymph node	T-cell lymphoma	8.10:1	10:1	*TRBC2*-restricted-mesenteric mass of lymphoma (Figure 9 panels A and B), with secondary involvement of the paracortex of an adjacent lymph node (Figure 9 panels C–F)
Cat 7	Siamese	F	4Y 9M	Nasopharynx mass	T-cell lymphoma	3.88:1	6:1	*TRBC2*-restricted

To estimate the degree of polymorphism in the coding regions of *TRBC1* and *TRBC2,* we also sequenced part of exon 1, the largest *TRBC* coding region exon, which is 387 bases long in both cat and dog. We sequenced a 299-base segment of cat *TRBC1* and *TRBC2* starting at position 100 of exon 1 of each isotype for 10 of our 20 cat DNA samples, and we compared the sequence data with the IMGT reference sequences (Accession number IMGT000037). We found SNPs in 4 of our 10 cat samples, with 1 cat showing 2 SNPs in *TRBC1* and 1 SNP in *TRBC2*, while the other 3 cats each possessed a single identical SNP in *TRBC2* (Appendix A, Table A2). Notably in the cat with SNPs in both *TRBC1* and *TRBC2*, one of the SNPs was shared between *TRBC1* and *TRBC2*. In summary, in the cat, similar levels of polymorphism are seen between the coding region and 3′ UTR.

**Figure A4:**
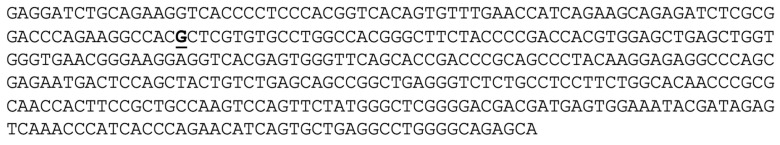
Our consensus dog TRBC1 exon 1 sequence. Exon 1 of dog TRBC1 (which we have submitted to Genbank as PZ103605). Position 84 in the IMGT reference sequence, BK065025, is given as A but is G (bold and underlined) in all reads in 100% of our samples. This polymorphism is synonymous and so does not affect the amino acid sequence.

**Figure A5:**
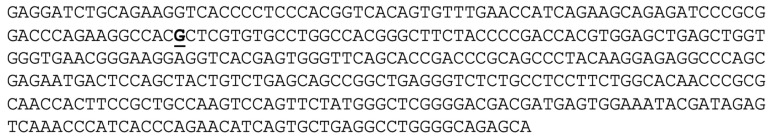
Our consensus dog TRBC2 exon 1 sequence. Exon 1 of dog TRBC2 (which we have submitted to Genbank as PZ103606). Position 84 in the IMGT reference sequence, HE653929, is given as A, but is G (bold and underlined) in all reads in 100% of our samples. This polymorphism is synonymous and so does not affect the amino acid sequence.

We sequenced a 292-base segment of dog *TRBC1* and *TRBC2* starting at position 3 of exon 1 of each isotype for all 10 of our dog DNA samples and compared the sequence data with the IMGT reference sequences (Accession numbers BK065025 and HE653929). We found no coding region polymorphisms in the dog, consistent with the lack of 3′ UTR SNPs. However, we demonstrated a single synonymous base difference in all of our dog *TRBC1* and *TRBC2* sequences, compared with the IMGT reference sequences (BK065025 and HE653929), summarized in Appendix A Figure A4 and Figure A5 (sequences submitted to NCBI Genbank as PZ103605 and PZ103606).

In summary, these results also demonstrated that any part of the 3′ UTR sequence could be used for probe design in dogs, but specific parts of the 3′ UTR sequence needed to be avoided in cats, in order to make probes applicable to any individual cat (Figure 4 and Figure 5).

### 3.3. Detection of Dog TRBC1 and TRBC2 in Benign Formalin-Fixed Paraffin-Embedded Tissue

**Figure 6:**
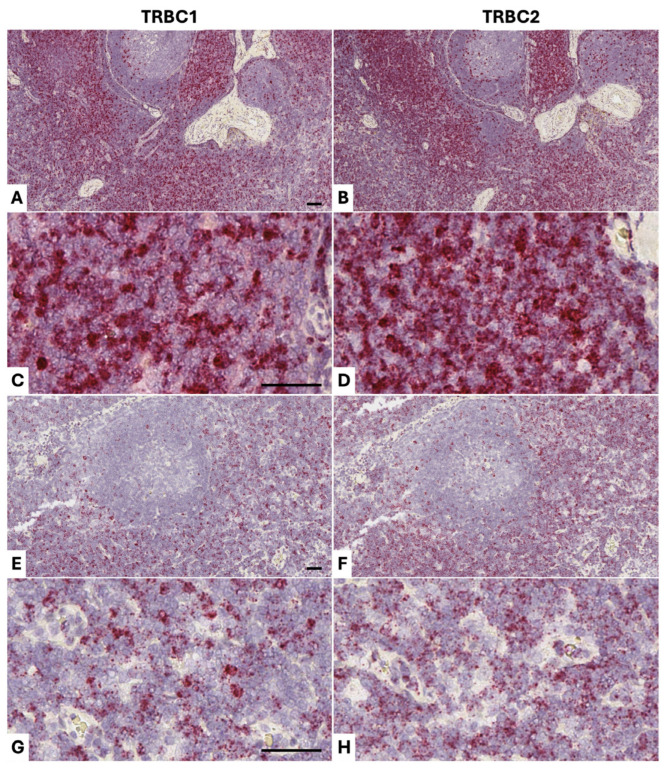
BaseScope^TM^ staining of dog benign lymphoid tissue (positive staining in red; nuclear hematoxylin counterstain in blue). A benign pharyngeal lymph node from a 12-year-old female springer spaniel (Dog 2), stained for *TRBC1* (**A**,**C**) and *TRBC2* (**B**,**D**), shows an approximately 1:1 *TRBC2*:*TRBC1* ratio of positive cells. A B-cell follicle is present in the center at the top of panels A and B and shows minimal staining, except in follicular helper T-cells, while there is extensive positivity among T-cells in the paracortex. A benign gastric lymph node from a 7-year-old male springer spaniel (Dog 3), stained for *TRBC1* (**E**,**G**) and *TRBC2* (**F**,**H**), shows an approximately 2:1 *TRBC2*:*TRBC1* ratio of positive cells. A B-cell follicle is present in the center of panels E and F, with a similar distribution of positively stained T-cells to the B-cell follicle in A and B. Panels G and H demonstrate variation in transcript levels (variation in numbers of dots/intensity of staining) between individual T-cells. Scale bars in all panels are 50 μm.

Since FFPE tissue represents the standard diagnostic sample type in veterinary pathology, we made BaseScope^TM^ probes specific to the 3′UTR of *TRBC1* and *TRBC2* and tested these on FFPE sections from benign lymphoid tissues from dogs (n = 5) (Figure 6; Table 1). As expected, multiple dots were seen in association with lymphocyte nuclei, each dot corresponding to one target RNA transcript. As this is a small proof-of-concept study and a larger study would be needed to determine a physiological normal range of *TRBC2*+: *TRBC1*+ cells, we chose to estimate this range in benign samples, much as practicing clinical pathologists do for the κ/λ light chain ratio in human clinical samples. In benign samples, there was some skewing towards *TRBC2*, with *TRBC2*+: *TRBC1*+ cell ratios ranging between 1:1 and 3:1, and this direction of skewing was corroborated by qPCR (Table 1). A similar preponderance of *TRBC2*+ over *TRBC1*+ cells is seen in humans [11,14].

**Figure 7:**
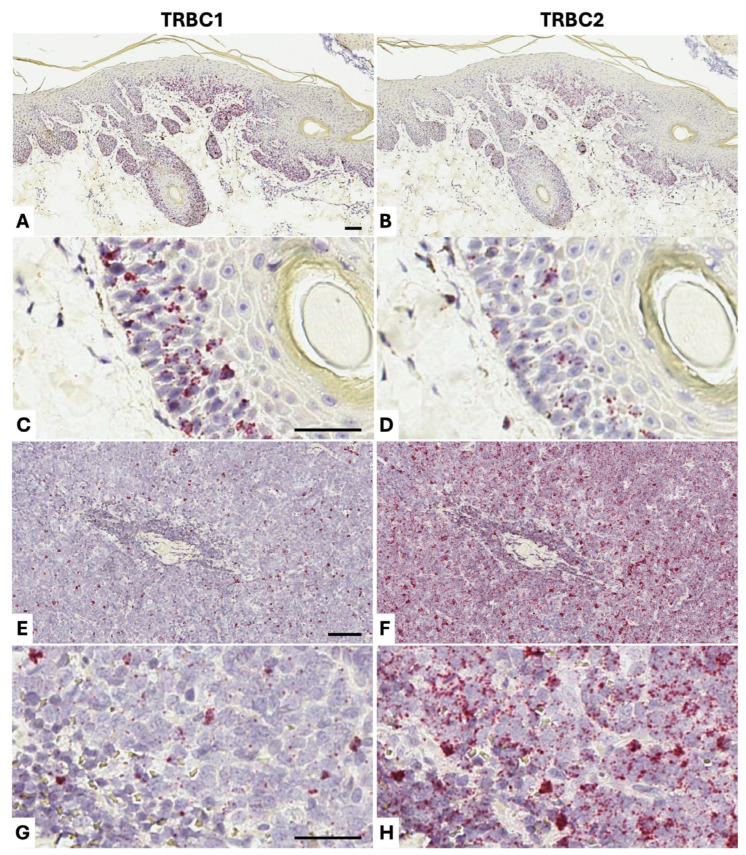
BaseScope^TM^ staining of dog T-cell lymphomas (positive staining in red; nuclear hematoxylin counterstain in blue). Skin from an 8-year-old female golden retriever (Dog 6) in which there is an epidermotropic T-cell lymphoma stained for *TRBC1* (**A**,**C**) and *TRBC2* (**B**,**D**), showing an approximate 5:1 ratio in favor of *TRBC1* (*TRBC2*:*TRBC1* = 0.2:1), i.e., a reversal of the physiological skewing. As expected in this diagnosis, T-cells are present between cells of the epidermis. A T-cell lymphoma in a mandibular lymph node from a 10-year-old male beagle (Dog 7) stained for *TRBC1* (**E**,**G**) and *TRBC2* (**F**,**H**) shows *TRBC2* restriction. In panels F and H, low-level *TRBC2* staining is present in the majority of cells, which are cells of the lymphoma, while higher levels of *TRBC2* expression can be seen in the scattered benign tumor-infiltrating lymphocytes. This is an excellent visual demonstration of the variability in transcript levels in individual cells, explaining why qPCR cannot be used to assess *TRBC2*:*TRBC1* ratios because it is confounded by variable transcript expression levels in individual cells. Scale bars in all panels are 50 μm.

To test the diagnostic utility of our approach, we applied *TRBC1/TRBC2* BaseScope^TM^ detection to two confirmed cases of canine T-cell lymphoma. Our method gave beautiful in situ visualization of the epidermotropic T-cells in a cutaneous T-cell lymphoma from an 8-year-old female golden retriever (Dog 6). In addition, it demonstrated *TRBC1* restriction of the lymphoma cells (Figure 7 panels A–D), which could obviate the need for PCR-based clonality testing (PARR), saving time and money and avoiding the need to send sample material to a specialist diagnostic center that can run the clonality testing. We stained a T-cell lymphoma in a mandibular lymph node from a 10-year-old male beagle (Dog 7), demonstrating *TRBC2* restriction (Figure 7). Relatively low level *TRBC2* expression is seen in the lymphoma cells, with higher levels of *TRBC2* expression in the scattered benign tumor-infiltrating lymphocytes. This variability in transcript levels in individual cells explains why qPCR cannot be used to assess *TRBC2*:*TRBC1* ratios accurately enough to comment on likely clonal status, because it is confounded by variable transcript expression levels in individual cells. Variable transcript expression levels also explain why qPCR appears to show skewing towards *TRBC1*, on a total transcript basis, while more of the T-cells in benign populations are *TRBC2*+. Some *TRBC1*+ cells visualized by BaseScope^TM^ show a higher staining intensity and/or number of dots than are seen in *TRBC2*-expressing cells. These results provide proof-of-concept for the potential use of *TRBC1* and *TRBC2* detection as a surrogate for clonal status in canine T-cell populations that are suspected to be T-cell lymphoma.

### 3.4. Detection of Cat TRBC1 and TRBC2 in Benign Formalin-Fixed Paraffin-Embedded Tissue

**Figure 8:**
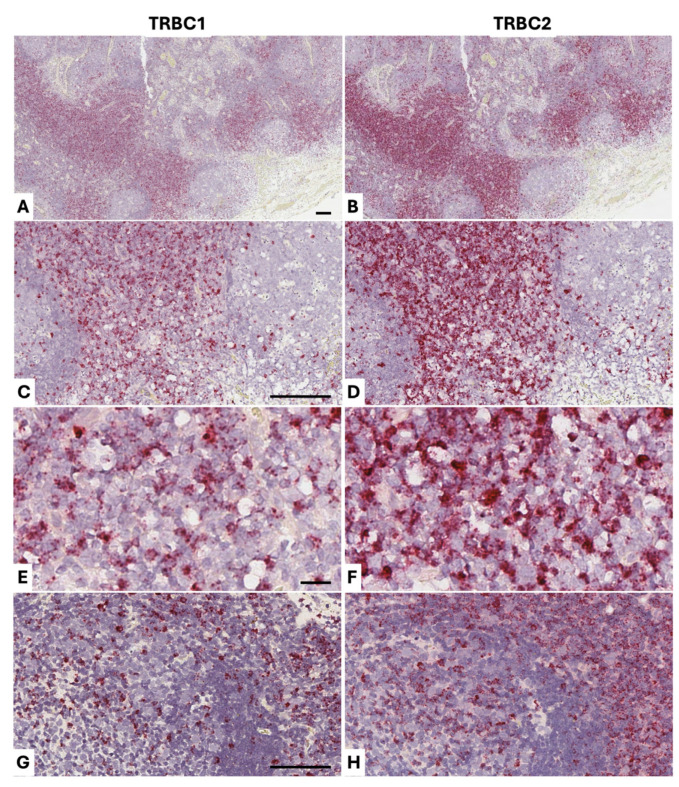
BaseScope^TM^ staining of cat benign lymphoid tissue (positive staining in red; nuclear hematoxylin counterstain in blue). A benign sternal lymph node from a 6-year-old male domestic shorthair (Cat 2), stained for *TRBC1* (**A**,**C**,**E**) and *TRBC2* (**B**,**D**,**F**), shows an approximately 2:1 *TRBC2*:*TRBC1* ratio of positive cells. Several B-cell follicles are seen in panels A and B, and there are B-cell follicles on the left and right sides of the images in C and D. The B-cells in the follicles are unstained for *TRBC1* and *TRBC2*, but there is positive staining of follicular helper T-cells, which are a mixture of *TRBC1*+ and *TRBC2*+. There is extensive *TRBC1* and *TRBC2* positivity in the paracortex, which is shown on high magnification in panels E and F, where the 2:1 *TRBC2*:*TRBC1* ratio is best appreciated. A benign sternal lymph node from a 6-year-old male domestic shorthair (Cat 2), stained for *TRBC1* (**G**) and *TRBC2* (**H**), shows an approximately 3:1 *TRBC2*:*TRBC1* ratio of positive cells. A B-cell follicle is present on the left side of the panels, with paracortex on the right side, and the distribution of *TRBC1*+ and *TRBC2*+ T-cells in these areas is similar to that described for panels A–F. These results were corroborated by qPCR results using RNA extracted from the FFPE tissue sections (Table 2). Scale bars in all panels are 50 μm.

We also made BaseScope^TM^ probes specific to the 3′UTR of feline *TRBC1* and *TRBC2* and tested these on FFPE sections from benign lymphoid tissues from cats (n = 5) (Figure 8; Table 1). As expected, multiple dots were seen in association with lymphocyte nuclei, each dot corresponding to one target RNA transcript. As with the dog samples, we chose to estimate the range of *TRBC2*+: *TRBC1*+ cell ratios in benign samples. In benign samples, there was some skewing towards *TRBC2*, with *TRBC2*+: *TRBC1*+ cell ratios ranging between 1:1 and 3:1, as in the dog, and this direction of skewing was corroborated by qPCR (Table 2).

**Figure 9:**
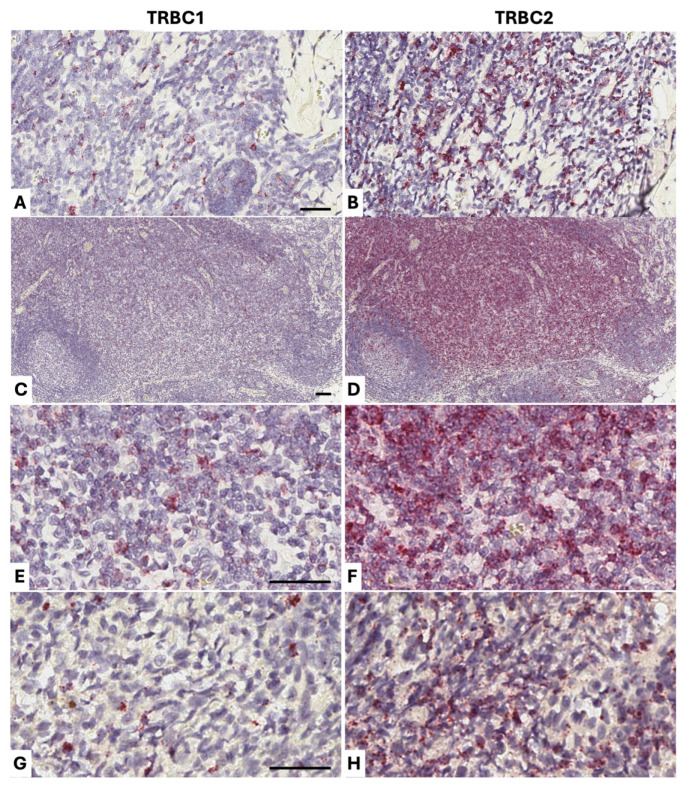
BaseScope^TM^ staining of cat T-cell lymphomas (positive staining in red; nuclear hematoxylin counterstain in blue). Mesenteric adipose from a 12-year-old Russian blue cat (Cat 6) which contains an infiltrate of medium-to-large T-cells that appear *TRBC2*-restricted (panels (**A**,**B**)). A lymph node present within the mesenteric adipose retains its B-cell follicles (bottom left hand part of panels (**C**,**D**)) but shows widespread paracortical expansion by a similar *TRBC2*-restricted population (shown at higher magnification in panels (**E**,**F**)), indicating spread of the T-cell lymphoma into the lymph node. (Figure 9 panels (**C**–**F**)). We also applied the BaseScope^TM^ method to nasopharyngeal tissue from a 4-year-old Siamese cat (Cat 7), demonstrating *TRBC2* restriction of the T-cell population (panels (**G**,**H**)). These results were corroborated by qPCR results using RNA extracted from the FFPE tissue sections (Table 2). Scale bars in all panels are 50 μm.

We also applied *TRBC1/TRBC2* BaseScope^TM^ detection to two confirmed cases of feline T-cell lymphoma (Figure 9). We stained mesenteric tissue from a 12-year-old Russian blue cat (Cat 6), which contained a T-cell infiltrate in mesenteric adipose tissue that appeared *TRBC2*-restricted (Figure 9 panels A and B). The included lymph node showed widespread paracortical expansion by a similar *TRBC2*-restricted population, indicating spread of the T-cell lymphoma into the lymph node (Figure 9 panels C–F). We also applied the BaseScope^TM^ method to nasopharyngeal tissue from a 4-year-old Siamese cat (Cat 7), demonstrating *TRBC*2 restriction of this T-cell population (Figure 9 panels G and H). These results were corroborated by qPCR results using RNA extracted from the FFPE tissue sections (Table 2).

These results confirm the technical feasibility of using BaseScope^TM^ detection in routine diagnostic FFPE tissue samples from cats and dogs in order to distinguish clonal (also known as monoclonal) from polyclonal T-cell populations. This in situ hybridization approach allows concomitant assessment of lesional architecture, cellular morphology and potentially immunophenotype on serial sections, with assessment for T-cell monotypia (*TRBC1*/*TRBC2* restriction).

## 4. Discussion

### 4.1. TCR Locus Conservation Allows Translation of a Novel Assay from Human to Cat and Dog

This study utilizes the conserved principle of dual *TRBC* isotypes seen across mammalian species [22], to develop a diagnostic approach to T-cell lymphoma, similar to that we have developed for human samples [11,14]. Most notably, while *TRBC1* and *TRBC2* coding regions remain nearly identical within and between species due to purifying selection maintaining protein function, their 3′ UTRs show striking intraspecies divergence but also interspecies conservation. This pattern where, for example, the feline *TRBC1* 3′ UTR resembles the canine *TRBC1* 3′ UTR more than the feline 3′ UTR *TRBC2* implies critical regulatory roles maintained through evolutionary constraint [22]. These 3′ UTR differences may confer distinct post-transcriptional regulation, potentially explaining functional differences observed in one study between *TRBC1*+ and *TRBC2*+ T-cells observed in human studies [32]. No developmental or regulatory bias favoring *TRBC1* over *TRBC2* has been described to date, but this interesting observation warrants further investigation.

### 4.2. Utility of TCR Sequencing for Accurate Cat and Dog-Specific Assay Design

Due to the necessity to have an exact match between BaseScope^TM^ probe sequences and target RNA transcript sequences, in order to obtain strong positive staining, we began this study by confirming the exact canine and feline *TRBC1* and *TRCB2* sequences and assessing any polymorphisms. While a number of polymorphisms were seen in cat *TRBC1* and *TRCB2* 3′UTR, constraining probe positions, neither *TRBC1* nor *TRBC2* showed 3′UTR polymorphisms in the dog. We did, however, identify a single-nucleotide polymorphism (A>G at position 84) in the coding regions of dog *TRBC1* and *TRBC2* (Appendix A, Figure A4 and Figure A5) when compared with IMGT reference sequences (BK065025 and HE653929, respectively). This synonymous polymorphism was identical between *TRBC1* and *TRBC2*, meaning that it has no impact on the protein produced, although it would need to be considered if one were to design in situ hybridization probes to this part of the coding region. We have submitted our *TRBC1* and *TRBC2* exon 1 sequences to NCBI Genbank, accordingly (accession numbers: Genbank Reference PZ103605 and PZ103606).

### 4.3. This Proof of Concept Is an Early Demonstration of Clinical Feasibility

Our study demonstrates the feasibility of using assessment of *TRBC1/TRBC2* expression patterns as a diagnostic assay for T-cell clonality in veterinary pathology. We confirmed the DNA sequences, lack of introns and level of polymorphism in cat and dog *TRBC1* and *TRBC2* permitting isotype-specific detection. In this proof-of-concept study, we established very approximate baseline expression *TRBC2*:*TRBC1* expression ratios in normal canine and feline tissues and showed that malignant T-cell populations display consistent *TRBC* isotype restriction. Studies using flow cytometry on human peripheral blood [33] and our own work on human tissue have demonstrated a *TRBC2*+ cell:*TRBC1* + cell ratio of around 1.2:1 [11,14]. This preliminary study suggests that the canine and feline *TRBC2*+ cell:*TRBC1*+ cell physiological ratios are between 1:1 and 3:1, meaning that they are not dissimilar to the human ratio. These findings provide proof-of-concept for a tissue-based diagnostic approach that could address current limitations in veterinary T-cell lymphoma diagnosis, analogous to the approach we have described for human clinical samples [11,14]. Compared with PCR-based clonality assays (PARR), a staining-based assessment of *TRBC* monotypia, as a surrogate for T-cell clonality, could provide morphological and immunophenotypic context. This is because serial sections can be stained with hematoxylin and eosin for *TRBC1*, *TRBC2* and other T-cell markers, meaning that it may be possible to determine whether the suspicious T-cell population is *TRBC1* or *TRBC2*-restricted. This could facilitate interpretation and avoid false positives [11,12,13,14]. Staining-based assays are also likely to be cheaper and can be performed in more local, less specialized centers. If specialist referral can be avoided, this should improve turnaround times and further decrease costs.

Inevitably, an RNA-based test will suffer from various limitations. RNA is relatively labile and, although a manual BaseScope^TM^ assay is available, an RNA-based test is best performed on an automated immunostainer to minimize RNA degradation. This may preclude the use of such a test in smaller laboratories that rely on manual staining. Good RNA preservation in tissue specimens is also critical, and this relies on careful pre-analytical handling of material, such as placing the specimen rapidly into neutral buffered formalin. Unfortunately, diagnostic laboratories have minimal control over this step in the tissue pathway.

### 4.4. Limitations of Current Study and Additional Work Required for Development of a Clinically Deployable In Situ Hybridization-Based Assay

While these results are promising, several limitations warrant consideration, and this should simply be regarded as a proof-of-concept study with very small cohort sizes, giving a descriptive rather than a quantitative output. Validation in larger, multi-center studies will be essential to develop standardized scoring criteria and diagnostic cut-off ratios. In particular, multi-center studies of larger cohorts will be required to establish the normal expression ratios for *TRBC1* and *TRBC2* across breeds, ages, geographic locations, anatomic sites, T-cell subsets and benign pathologies for both cats and dogs. Such studies will enable determination of diagnostic cut-offs for the ratio of *TRBC1*+ cells to *TRBC2*+ cells by determining the range of *TRBC2*+ cell:*TRBC1* + cell ratios in benign T-cell populations. In addition, a broad range of T-cell lymphomas of different histological subtypes and from different anatomical locations will need to be stained in order to be certain that *TRBC1* or *TRBC2* restriction, or monotypia, is seen in the majority of T-cell lymphomas, because a proportion may be *TRBC1*- and *TRBC2*-, while occasional lymphomas may be positive for both.

In some tissue sections, high numbers of positive dots, each corresponding to one or a small number of *TRBC* transcripts, were seen. In some areas, there were such large numbers of dots that it was difficult to determine which nucleus the detected transcripts were likely to be associated with. A subsequent study will need to optimize this level of staining in *TRBC1*+ or *TRBC2*+ cells in one of two ways. Firstly, one might decrease the BaseScope^TM^ signal amplification by, for example, shortening the incubation time for the tyramide amplification step (Amp7). Secondly, one might decrease the numbers of variably overlapping nuclei in each histological section by cutting the sections thinner than 3.5 µm, for example, at 1–2 µm. Additionally, duplex *TRBC1*/*2* assays or duplex assays with CD4 or CD8 may facilitate interpretation of BaseScope^TM^ staining results [14]. It is clear, however, in the tissue stained in this study that there is considerable variation in the numbers of transcripts in cells, which, when it occurs in adjacent cells, most likely reflects real biological variation, rather than technical artefact, which would affect the whole section. Whether variation in *TRBC* transcript level is a dynamic process related to activation status or other effects of the interactions with adjacent cells or is determined by T-cell subtype remains to be seen, and a larger study will be needed to investigate this. Without isotype-specific reagents for the detection of the TCRbeta1 and TCRbeta2 proteins in cats and dogs, it is not clear whether these variations in transcript level translate into differences in levels of the corresponding proteins.

### 4.5. Considerations of Alternative Approaches to Determining the TRBC2+ Cell:TRBC1 + Cell Ratio

One might consider determining the *TRBC2*+ cell:*TRBC1* + cell ratio in other ways— for example, by making anti-TCRbeta1/2-specific antibodies. While the very high (>99%) amino acid level identity between TCRbeta1 and TCRbeta2 proteins in each species might make generation of isotype-specific anti-TCRbeta1/2 antibodies difficult in the cat and dog, the fact that we have achieved this in an analogous situation for human TCRbeta1/2 [11,14] raises the possibility of doing so in other species. In general, if relevant antibody generation is achievable, an immunohistochemical assay is preferred, as it is less sensitive to pre-analytical tissue handling, cheaper and often easier to interpret histologically.

We also considered the utility of a qPCR-based assay to determine the *TRBC2*:*TRBC1* transcript ratio. However, the qPCR-derived ratio is substantially confounded by the transcript expression level in individual cells, as can be seen in Figure 6 (panel D), making qPCR unsuitable for this purpose. *TRBC* restriction must be assessed in terms of the ratio of numbers of cells that are positive for each isotype (*TRBC1* or *TRBC2*), rather than the ratio of total extracted *TRBC1* and *TRBC2* transcripts.

### 4.6. Potential Biological Limitations of an Assay for T-Cell Monotypia

The greatest diagnostic utility of *TRBC* restriction might be in distinguishing truly neoplastic clonal expansions from small reactive T-cell clones that can occur in inflammatory conditions, for example, in canine monocytic ehrlichiosis, a tick-borne disease in dogs caused by the bacterium *Ehrlichia canis* [7]. Clonal T-cell expansion in this condition can cause false positive results when using the standard PCR-based veterinary clonality assay, PARR [13,34]. Assessment of *TRBC* restriction by means of *TRBC1/2* BaseScope^TM^ CISH could be very helpful in this context, because it maintains architectural and morphological context of any *TRBC*-restricted (and thus presumed clonal) T-cell population. It could also permit comparison between sections stained for *TRBC1/2* with serial sections immunostained with relevant T-cell markers [11,12,14]. Thus, false positive diagnoses of T-cell lymphoma might be avoided. Notwithstanding, larger studies are essential to determine whether *TRBC1* or *TRBC2* skewing, which might raise concern for T-cell lymphoma, can be seen in non-neoplastic lymphocytic infiltrates, such as reactive inflammatory processes and autoimmune or allergic infiltrates.

### 4.7. Potential Therapeutic Utility of TRBC1 and TRBC2 Targeting

Beyond diagnostic applications, the existence of *TRBC* isotypes has led to the possibility of their use as therapeutic targets. CAR-T-cell strategies targeting specific *TRBC* isotypes have shown promise in human clinical trials, with anti-TCRbeta1 CAR-T-cells demonstrating efficacy against T-cell malignancies while preserving TCRbeta2+ normal T-cells [35]. This selective targeting approach is feasible because approximately 50% of normal T-cells would remain intact, potentially maintaining immune function while eliminating clonal populations. The successful identification of *TRBC* isotype restriction in our veterinary lymphoma cases suggests that therapeutic strategies targeting clonal populations could potentially be developed for companion animals. The assay developed in this study may act as a companion diagnostic test.

## 5. Conclusions

This proof-of-concept study establishes *TRBC1*/*TRBC2* expression analysis as a possible diagnostic approach for T-cell lymphoma in veterinary pathology. By allowing assessment of the architecture, cytomorphology and potentially, on serial sections, the immunophenotype of a T-cell infiltrate, this in situ hybridization method might address key limitations of current PCR-based clonality assessment (PARR) while offering the potential for routine implementation in diagnostic laboratories. If subsequent larger studies indicate that the test performs well enough to be introduced into clinical diagnostic practice, it has the potential to reduce cost and to obviate the need to refer material to more specialist laboratories and improve diagnostic turnaround times.

## Data Availability

The data presented in this study are openly available in Genbank (https://www.ncbi.nlm.nih.gov/genbank/), reference numbers PZ103605 and PZ103606.

## Abbreviations

The following abbreviations are used in this manuscript:

**Abbreviation**	**Meaning**
BLAST	Basic Local Alignment Search Tool
bp	Base pairs
CAR	Chimeric antigen receptor
CDS	Coding sequence
CISH	Chromogenic in situ hybridization
FFPE	Formalin-fixed paraffin-embedded
IMGT	International ImMunoGeneTics information system
PARR	PCR for Antigen Receptor Rearrangements
PCR	Polymerase chain reaction
qPCR	Quantitative PCR (also called Real-Time PCR)
RAG-1	Recombination activating gene 1
RAG-2	Recombination activating gene 2
Snp	Single nucleotide polymorphism
TCR	T-cell receptor
TdT	Terminal deoxyribonucleotidyl transferase
*TRBC*	Gene segment or part of RNA transcript encoding the constant region of T-cell receptor beta
UTR	Untranslated region

## Appendix A

**Table A1:** NCBI accession codes of database-derived sequences used in consensus assembly sequences for cat and dog TRBC1 and TRBC2.

Species	Gene	Accession Number	Sample Origin
Dog	*TRBC1*	DN352916.1	est
		DN270394.1	est
		DN408913.1	est
		C0618951.1	est
		DN408268.1	est
		DN352987.1	est
		DN352613.1	est
Dog	*TRBC2*	D16410.1	nr
		DN371162	est
		GR891931	est
		HE653929.1	nr/nt
		CK999712	est
		BU749617	est
		DN353320	est
		CX000222	est
		CX000223	est
		DN354100	est
		NC_006598.3	nr/nt
		DN351945	est
Cat	*TRBC1*	NC_018724.2	nr/nt
		NT_203348.1	nr/nt
		AANG03031952_1	wgs
		ACBE01534668_1	wgs
		AANG03050173_1	wgs
Cat	*TRBC2*	SRR3218716	SRA
		NC_018724.2	nr/nt
		XR_002146559.1	nr/nt
		XR_002146558.1,	nr/nt
		SRR835503	SRA
		ERR1331678	SRA

**Table A2:** Cat TRBC1 and TRBC2 variant alleles.

Reference	Position (According to IMGT Exon 1 Reference Sequence)	IMGT Exon 1 Reference Sequence Base	Variant Allele Base	Read Count Containing Variant Allele	Total Read Count for Isotype	Variant Allele Frequency	Sample
*TRBC1*	201	C	T	136	955	0.1424	B3
*TRBC1*	209	G	A	88	832	0.1058	B3
*TRBC2*	144	T	C	6620	7898	0.8382	A51
*TRBC2*	144	T	C	5248	7989	0.6569	A37
*TRBC2*	144	T	C	516	3051	0.1691	C45
*TRBC2*	201	C	T	164	1024	0.1602	B3
